# Biodiversity in and around Greenhouses: Benefits and Potential Risks for Pest Management

**DOI:** 10.3390/insects12100933

**Published:** 2021-10-14

**Authors:** Gerben J. Messelink, Jérôme Lambion, Arne Janssen, Paul C. J. van Rijn

**Affiliations:** 1 BU Greenhouse Horticulture, Wageningen Research, Violierenweg 1, 2665 MV Bleiswijk, The Netherlands; 2 Laboratory of Entomology, Wageningen University, Droevendaalsesteeg 1, 6708 PB Wageningen, The Netherlands; 3 Groupe de Recherche and Agriculture Biologique (GRAB), Maison de la Bio 255, Chemin de la Castelette, 84911 Avignon, France; jerome.lambion@grab.fr; 4 IBED, Department Evolutionary and Population Biology, Institute for Biodiversity and Ecosystem Dynamics, University of Amsterdam, Science Park 904, 1098 XH Amsterdam, The Netherlands; arne.janssen@uva.nl (A.J.); P.C.J.vanRijn@uva.nl (P.C.J.v.R.); 5 Department of Entomology, Federal University of Viçosa, Viçosa 36570-900, Minas Gerais, Brazil

**Keywords:** biological control, ecosystem services, functional agrobiodiversity, plant diversity, parasitoids, predators, protected cultivation

## Abstract

**Simple Summary:**

The role of plant diversity near greenhouses is heavily debated because it may have both negative and positive effects on pest control inside greenhouses. In this review, we discuss these potential risks and benefits. Although there is the risk of an increased influx of some pests and of viruses transmitted by pests, we argue that biodiversity in the adjacent environment usually has limited effects on pest abundance in greenhouses in temperate climates, as most greenhouse pests in temperate climates are of exotic origin. The main benefit of increased biodiversity near greenhouses is the immigration of natural enemies that can suppress pests inside greenhouses. An open question is how this can be promoted by specific plant communities, plant characteristics, and habitats while minimising risks. Plant biodiversity inside greenhouses can also support biological control. We summarise general methods that growers can use to enhance pest control with functional biodiversity and suggest that it is particularly important to study how biodiversity inside and outside greenhouses can be linked to enhancement of biological pest control with both released and naturally occurring species of natural enemies.

**Abstract:**

One of the ecosystem services of biodiversity is the contribution to pest control through conservation and stimulation of natural enemies. However, whether plant diversity around greenhouses is beneficial or a potential risk is heavily debated. In this review, we argue that most greenhouse pests in temperate climates are of exotic origin and infest greenhouses mainly through transportation of plant material. For indigenous pests, we discuss the potential ways in which plant diversity around greenhouses can facilitate or prevent pest migrations into greenhouses. As shown in several studies, an important benefit of increased plant diversity around greenhouses is the stimulation of indigenous natural enemies that migrate to greenhouses, where they suppress both indigenous and exotic pests. How this influx can be supported by specific plant communities, plant characteristics, and habitats while minimising risks of increasing greenhouse pest densities, virus transmission, or hyperparasitism needs further studies. It also requires a better understanding of the underlying processes that link biodiversity with pest management. Inside greenhouses, plant biodiversity can also support biological control. We summarise general methods that growers can use to enhance pest control with functional biodiversity and suggest that it is particularly important to study how biodiversity inside and outside greenhouses can be linked to enhancement of biological pest control with both released and naturally occurring species of natural enemies.

## 1. Introduction

**Figure 1:**
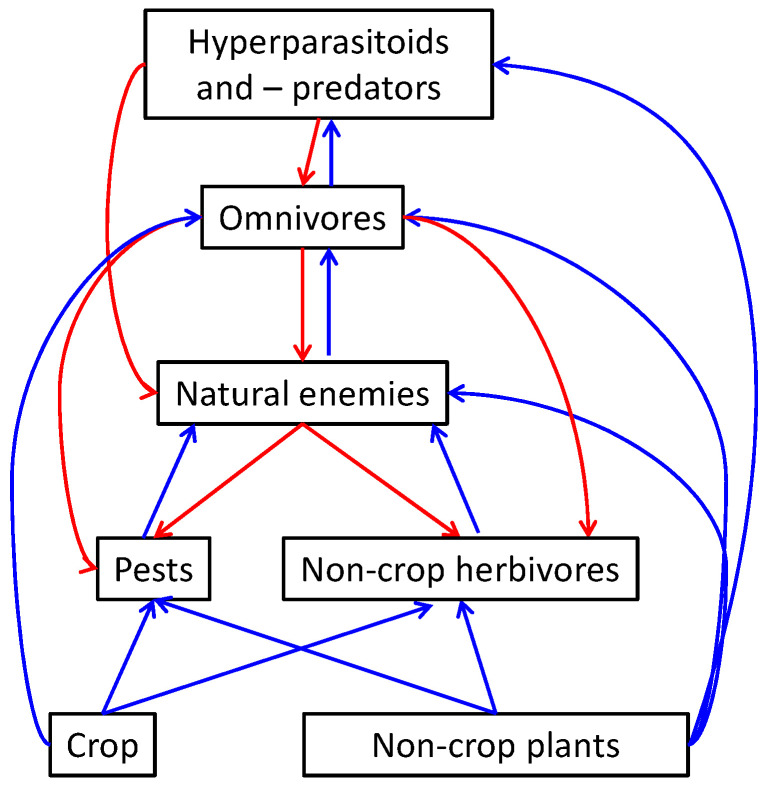
Possible direct and indirect effects of plant biodiversity on the assemblage of pests and other arthropods (after Gurr et al. 2003) [9]. Blue lines represent positive, and red lines negative effects. Combinations of direct effects yield indirect effects (not shown here).

Biodiversity in general increases the level and stability of ecosystems services, which is defined as a suite of benefits that ecosystems provide to humanity [1] (see also The Intergovernmental Science-Policy Platform on Biodiversity and Ecosystem Services, IPBES). Pollination by insects is one these ecosystem services that is essential for the production of many agricultural crops [1]. Another ecosystem service is the contribution to pest control through conservation and stimulation of natural enemies [2]. Increasing natural enemy richness in general enhances pest control [3], and plants and plant diversity can increase natural enemy richness and densities by providing them with alternative prey/hosts, pollen, nectar, and refugia [4]. Several studies have indeed shown enhanced pest control through increased plant diversity in or near cropping systems [4,5,6,7]. A meta-analysis of biodiversity experiments in relation to pest management shows overall increased natural enemy densities and enhanced pest control in diversified crops than in crops with fewer or no other plant species [8], although this did not hold for each individual study [1,8]. There are several potential underlying processes of direct and indirect interactions that determine the direction and magnitude of the effects of plant diversity on pest suppression (Figure 1, adapted from Gurr et al. [9]).

Plant diversity can enhance pest control through positive effects on natural enemies, but it can also increase densities of omnivores or hyperparasitoids, which can disrupt pest control by the natural enemies [10,11]. Moreover, certain plant species can also increase pest diversity and densities [8]. Not only plant species composition but also landscape composition and configuration have a major impact on pollination and natural pest control [12]. Natural enemies generally show a strong positive response to increased landscape complexity, but again, many studies show that this does not necessarily enhance pest control [13,14]. Thus, the impact of biodiversity on pest control is not straightforward, and a better understanding of the underlying ecological processes that link biodiversity with pest management is required to improve agricultural production systems. This need for more knowledge is particularly true for greenhouse crops and their interaction with their environment. In contrast to open field crops, very little is known about the role of biodiversity around greenhouses on pest control and pollination in greenhouse crops. Nowadays, there are many initiatives to increase plant diversity in urbanized areas, including greenhouse surroundings, instigated by the recent awareness of the strong insect declines worldwide [15,16]. The impact of such changes of the surroundings on the abundance of pests in greenhouses is not well known yet. Better protection against the influx of pests is often seen as one of the benefits of horticulture in greenhouses compared to outdoor crop cultivation. This protection will depend on the greenhouse structure, which varies from rather basic plastic tunnels to high-tech glasshouses [17]. Some of these modern glasshouses are provided with insect gauze in the ventilation windows, which reduces the interchange of arthropods with the environment. However, such adjustments to ventilation windows are costly and reduce the ventilation capacity and are therefore not widely applied [17]. Thus, in most cases, greenhouses are connected to the environment through the ventilation windows. This open connection with the environment is reason of concern about the increase of plant diversity near greenhouses by some growers because it would potentially increase the influx of pests and viruses from the surrounding vegetation. In fact, many growers prefer short lawns consisting of one or a few grass species near their greenhouses because they think it reduces the risk of pest influx. Here, we analyse whether there are indeed such risks of increased pest influx through increased plant diversity, and we discuss whether such biodiversity can actually be used to enhance greenhouse pest management.

## 2. Where Do Pests in Greenhouses Come from?

Most greenhouse pests, particularly in temperate climates in Northern Europe, are of exotic origin (Table 1) and colonize greenhouses through the transport of plant material such as cuttings, seedlings, and potted plants. For example, western flower thrips, *Frankliniella occidentalis* Pergande (Thysanoptera: Thripidae), originates from western North America but became a worldwide pest through its establishment in greenhouses [18], and there have been many more cases where greenhouses in fact facilitated the spread of exotic pests around the world [19,20,21]. Among the exotic pests in Northern European greenhouses, many originated from the Mediterranean area (Table 1). These pests migrate or are introduced to northern countries during hot summers, but they can often overwinter and establish local populations in greenhouses, as is the case, for example, for the Southern green stink bug *Nezara viridula* (Linnaeus) (Heteroptera: Pentatomidae). Some exotic pest species are rather host-plant specific and will not easily find suitable host plants outside greenhouses, for example, specific orchid pests (Table 1).

Others are polyphagous and can establish on vegetation outside greenhouses; however, most pests with a tropical or subtropical origin are not likely to survive winters in the temperate climate zone. In Dutch greenhouses, for example, at least eight exotic thrips species are established in various crops (Table 1), but only a few were found outside greenhouses and only in limited numbers [24]. Obviously, this may change due to global warming, as has already been observed for some exotic pests [25]. Yet, in general, it can be assumed that most pests in greenhouses in temperate climates did not enter greenhouses from the surrounding vegetation, but originate from cross-contamination among greenhouses. However, once established in greenhouses, some exotic pests can use outdoor vegetation to spread to other greenhouses during summer, which has been observed for exotic moths, such as *Tuta absoluta* (Meyrick) (Lepidoptera: Gelechiidae) (authors, personal observations). In warmer climatic zones, the situation is different because more pest species overwinter in the natural environment and the greenhouse structures, such as plastic tunnels, are more open than glasshouses in temperate regions.

**Figure 2:**
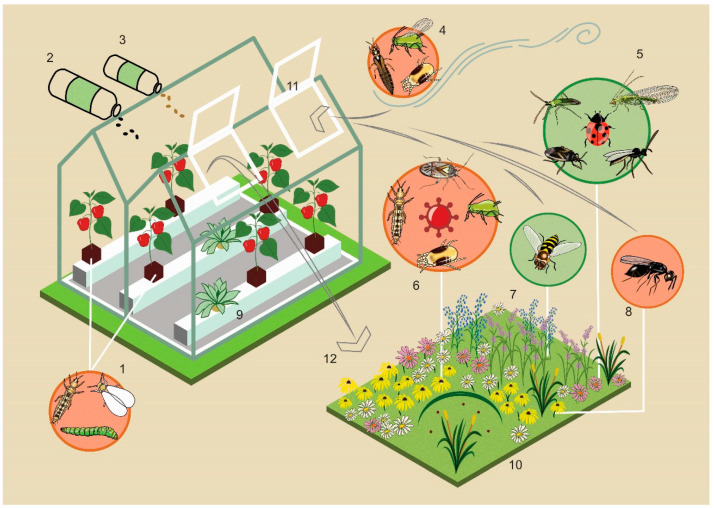
Schematic overview of potential pathways of pests, viruses and hyperparasitoids (orange), natural enemies and pollinators (green), and the role of plant diversity in and around greenhouses in these pathways. Numbers indicate: (1) unintentional introductions of exotic pests through plant material; (2) introduction of mass-produced natural enemies; (3) provision of food for natural enemies; (4) influx of indigenous pests through long-distance aerial dispersal; (5) indigenous natural enemies around greenhouses that control pests outside and inside greenhouses; (6) indigenous pests around greenhouses that actively migrate into greenhouses and transmit viruses in some cases; (7) indigenous syrphids that migrate to greenhouses and contribute to pollination and aphid control inside greenhouses; (8) hyperparasitoids that migrate to greenhouses and disrupt biological control; (9) banker plants inside greenhouses that support natural enemies; (10) diversity of plant species near greenhouses that support natural enemies by providing shelter, nectar, alternative prey, and pollen; and (11) ventilation windows as entrances for indigenous pests and natural enemies but also (12) a possible pathway for migration of greenhouse pests and natural enemies to the vegetation near greenhouses.

The limited numbers of indigenous pests that enter greenhouses (Table 1) do so through aerial dispersal and to a lesser extent from the adjacent vegetation (Figure 2). In temperate climates, these indigenous species include some thrips, plant-feeding bugs, aphids, spider mites, and moths (Table 1). The most obvious way for arthropods to enter a greenhouse is through the ventilation windows. The contribution of the surrounding vegetation to this influx might be limited for cases of pests that are able to travel long distances on air currents, and this is the case for several small insect and mite pests. Thrips, for example, can travel distances of even hundreds of kilometres [26]. Most species of thrips outside greenhouses fly only during a few weeks per year, which can result in spectacular mass flights [27]. This phenomenon is well known for the grain thrips *Limothrips cerealium* Haliday (Thysanoptera: Thripidae) [27], a species that is harmless for greenhouse crops. Grain thrips also enter greenhouses during these mass flights and often confuse growers when found in high densities on sticky traps (Messelink, personal observations). Thrips migrate passively with the wind and are mostly found at a height of 6 meters [28], which is exactly the height of the ventilation windows of modern greenhouses. Thrips species that are pests in greenhouses, such as the European flower thrips *Frankliniella intonsa* (Trybom) (Thysanoptera: Thripidae), also have such mass flights, which have been observed in autumn at the end of the flowering season of wildflowers [29]. Other tiny pest species, such as aphids and spider mites, also use wind to migrate over long distances. Spider mites can become airborne by hanging down on a silken thread from higher positions, where the silk may be caught in turbulence which breaks the line [30]. This so-called “ballooning dispersal” enables spider mites to migrate over long distances. Spider mites can also migrate in groups. At high densities, when plants are overexploited, individuals gather at the plant apex to form a collective silk ball, and this structure can also be dispersed by wind [31]. Aphids disperse as winged morphs (alates) that are produced as a response to crowding or reduced host-plant quality [32], and those alates can travel long distances by wind. Aphid populations in temperate climates usually show a very clear migration peak in early spring and late autumn [33], and most greenhouse crops become infested by aphids in early spring. In summary, long-distance aerial dispersal is very common for pests like thrips, spider mites, and aphids and may also contribute to the influx of these pests into greenhouses. Removing vegetation around greenhouses with the purpose to reduce the influx of these pests may therefore not be effective. Other, possibly more important, sources of infestation are workers who carry these tiny insects attached to their clothes and hair into the greenhouse [34] and the introduction of nursery plants and other materials.

## 3. Potential Risks of Plant Biodiversity around Greenhouses

Despite the arguments developed above, plant biodiversity around greenhouses can also be a source of pests of crops inside greenhouses (Figure 2). Many greenhouse pests are polyphagous [35] and can survive and reproduce on a wide range of plant species potentially occurring around greenhouses. Thus, the presence of some of these host plants for greenhouse pests near greenhouses may potentially increase pest populations, which can subsequently migrate to the greenhouse crops. However, it is hardly known to what extent this actually occurs and by which pest species. In the Mediterranean greenhouse area in Almeria, western flower thrips and tobacco whiteflies have similar abundance patterns in horticultural crops within greenhouses and on outdoor perennial plants [36]. Moreover, the pests occurred for a shorter period on perennial plants than in the greenhouse crops and occurred later outdoors than indoors, suggesting that the perennial outdoor plants were not the initial pest source [36] but possibly became infested with pests from the greenhouse. Some specific plant species near greenhouses may be attractive for pest species that can subsequently migrate to greenhouse crops. Flowering roadsides are popular for their aesthetic value and ecosystem function for bees and other pollinators, but the pollen of these flowers are often also good food sources for many thrips species [37]. A recent study with flowering plants near greenhouses indeed showed that thrips densities increased compared to the densities in common and monthly mown weeds near greenhouses, but this did not result in higher pest densities inside the greenhouses [38]. Some plants, such as alfalfa, sunflowers, and chamomile, are attractive plants for tarnished plant bugs (*Lygus* spp.) [39,40] and may increase densities of these pests when planted near greenhouses. Some flowering plants can also provide food to moth pest species. In some cases, the presence of flowering plants indeed increased caterpillar damage in neighbouring outdoor crops [41,42], but in most cases, it is not clear whether increasing the attractiveness of flora for pest species near greenhouses does result in a higher pest influx of pests into greenhouses. The same plant diversity may also increase biological pest control outside greenhouses or retain pests outside greenhouses, which will be discussed below (see benefits section).

Besides pests, vegetation around greenhouses may also harbour plant viruses that are transmitted by insects, which can also be a risk for greenhouse crops. The best-known and most widespread plant virus is the Tomato Spotted Wild Virus (TSWV), which can infect almost 1100 plant species [43], including many weeds that are common near greenhouses [44]. Because these host plants are so common, any further diversification of the flora would probably not change the potential reservoir of TSWV-infected plants very much. TSWV is known to be transmitted by eight species of thrips, including western flower thrips, the onion thrips, and the European flower thrips [45], of which the latter two can be abundant in flowers near greenhouses [24]. Almost all aphid species can transmit plant viruses belonging to the genera *Potyvirus, Luteovirus, Cucumovirus,* and *Closterovirus* [46]. Yet, apart from the cucurbits [47], most greenhouse crops do not suffer from aphid-transmitted viruses. The tobacco whitefly is notorious for vectoring the Tomato Yellow Leaf Curl Virus (TYLCV) and other viruses to tomato crops [48]. TYLCV is one of the most devastating viral diseases of tomato crops in tropical and temperate areas worldwide, including Mediterranean greenhouse areas. Thus, invasions of greenhouse crops by tobacco whiteflies from outside can be a serious risk for TYLCV transmissions when host plants of this virus around greenhouses are infected by both the virus and tobacco whiteflies. Recent studies in the Mediterranean area show that more closed greenhouses experience reduced TYLCV infections [49]. Increasing the suppression of virus vectors outside greenhouses may also reduce the infections of greenhouses by viruses.

## 4. Benefits of Biodiversity around Greenhouses

### 4.1. Contributing to Pest Control and Pollination inside Greenhouses

The most important contribution of biodiversity around greenhouses might be the control of pests inside greenhouses by natural enemies that originate from the surrounding vegetation. In outdoor crops, many studies indicate the potential of non-crop habitats for pest control [50], but how such habitats would affect pest control in greenhouses is hardly known. Although the potential influx of natural enemies in greenhouses is not well documented, there are several examples indicating that this may play a major role in biocontrol, particularly in warmer climates, where interchanges of pests and their enemies with outdoors is more common than in colder climates [51]. A recent study in northern China shows that a mix of six flowering plant species around greenhouses increased predator densities and aphid control on egg plants inside a greenhouse [38]. Tomato greenhouse crops in Mediterranean countries are often colonized by mirid predatory bugs, which are important for pest control in greenhouses [52,53,54,55]. An experiment in the south of France showed that this influx could be increased by planting marigold plants near and inside greenhouses, thus attracting and arresting these predators [56]. Sweet pepper crops in greenhouses in northern Italy are often colonized by predators of the genus *Orius* (Hemiptera: Anthocoridae), which are important for the control of thrips [57]. An extensive recent study in French strawberry greenhouses showed that various species of parasitoids that entered the greenhouses from the surroundings had a large contribution to aphid control [58]. The influx of natural enemies in greenhouses becomes even more interesting when these natural enemies are not commercially available for augmentative biological control, for example, because of the high costs for mass rearing. This is the case for the Mediterranean parasitoid *Necremnus tutae* Ribes & Bernardo (Hymenoptera: Eulophidae), which is extremely valuable for the control of the invasive South American tomato pinworm *T. absoluta* [59]. Parasitism by non-released species is also rather common in Northern European greenhouses, for example parasitism of caterpillars of the Golden twin-spot moth *Chrysodeixis chalcites* (Esper) (Lepidoptera: Noctuidae) by *Microplitis spinolae* (Nees) (Hymenoptera: Braconidae) and *Cotesia vanessae* (Reinhard) (Hymenoptera: Braconidae) [60]. Furthermore, the indigenous leaf miner parasitoid *Dacnusa siberica* Telenga (Hymenoptera: Braconidae) appeared to be an effective natural enemy of the invasive American serpentine leaf miner *Liriomyza trifolii* (Burgess) (Diptera: Agromyzidae) [61]. These examples also show that indigenous natural enemies from greenhouse surroundings can be important for the control of exotic pest species. Leaf miners in Northern European greenhouses are also often spontaneously parasitized by parasitoids from outside greenhouses [62].

The vegetation and the alternative hosts/prey in it will have a big impact on the influx of natural enemies into nearby greenhouses. Non-pest herbivores on non-crop plants can also indirectly interact with greenhouse pests when sharing the same species of natural enemies. This indirect interaction, called “apparent competition,” can enhance biological control, as shown in many studies [63]. The vegetation adjacent to greenhouses might even be more important for natural enemies than for pests because the latter often travel long distances by wind, as discussed above. In contrast, natural enemies are not known to passively travel such long distances. Some parasitoids can travel up to 15 km per day, but these daily distances go down to 4 km per day for mated and ovipositing females [64]. Parasitoids need nectar sources for their flight energy and to increase their rates of parasitism [65] and providing this nectar can induce medium- or long-range dispersal by parasitoids [66]. Thus, plants that provide nectar may act as fuel stations for dispersing parasitoids and most likely promote the influx of parasitoids into greenhouse crops.

Increasing numbers of parasitoids near greenhouses might also increase the numbers of hyperparasitoids, which are the enemies of pest enemies (Figure 1) [11]. Like parasitoids, hyperparasitoids can profit from the nectar in flowering plants [67], which may increase their densities and eventually the levels of hyperparasitism in greenhouses and thereby disrupt pest control. Studies in French strawberry greenhouses showed higher rates of hyperparasitism in open than in closed greenhouses [58], thus showing a potential risk of more hyperparasitism when greenhouses are better connected with their environment. However, aphid hyperparasitoids were already observed in Dutch greenhouses very early in the season, suggesting that they overwinter in greenhouses [68]. In that case, the wild vegetation near greenhouses might have less impact on the level of hyperparasitism in greenhouses. The type of vegetation around greenhouses can also affect the risk of hyperparasitoid influx into greenhouses. A Chinese study, for example, showed that higher proportions of woodland near greenhouses resulted in lower levels of hyperparasitism of aphid parasitoids compared to surroundings with more cropland [69]. Stimulating aphid predators in wild flora with specific plant species [70] may also be a way to reduce aphid mummies in wild vegetation and thereby also reduce the risk of aphid hyperparasitoids.

The flora around greenhouses might not only facilitate the influx of natural enemies but also that of pollinators, such as wild bees and syrphids. This is particularly useful for soft fruit crops, where pollination by commercial bumblebees is not always effective [71]. It is even more useful when some of these wild pollinators also function as biological control agents of aphids, as some species of syrphids do [72]. Influx of wild pollinators may also be useful for providing species that are active during different times of the year and at different temperatures.

Hence, overall, we can conclude that several studies indicate the importance of natural enemies and pollinators originating from greenhouse surroundings for pest management and pollination in greenhouses, and these ecosystem services might be enhanced by adapting and optimizing the flora and habitats around greenhouses.

### 4.2. Reducing Pest Densities outside Greenhouses

We argued that many pests in greenhouses are of exotic origin and are permanently established in greenhouses and that some indigenous pests that enter greenhouses through ventilation windows travel long distances. Thus, aiming to control greenhouse pests in the surroundings of greenhouses in order to decrease the influx of greenhouse pests is not relevant in many cases or is expected to have limited impact. However, it might be essential for some pests that do not travel long distances and that are difficult to control with augmentative biological control inside greenhouses. For example, this is the case for tarnished plant bugs of the genus *Lygus* (Hemiptera: Miridae). Only adults of this pest migrate to greenhouse crops, where they can survive for long periods and cause serious crops damage [73]. Reducing the influx of adult plant bugs might be achieved by promoting parasitism or predation of the nymphal stages in the vegetation near greenhouses [74]. In this way, increasing biodiversity and re-create complex ecosystems around greenhouses may reduce densities of outdoor pests that can potentially infect greenhouse crops. This approach might also be relevant in warmer climates where the influx of greenhouse pests, such as whiteflies and thrips, from the greenhouse surroundings is much higher than in temperate climates. Studies in Spain showed that certain native plants near greenhouses, such as bolina (*Genista umbellata* Poir), joint pine (*Ephedra fragilis* Desf.), and cambrón (*Lycium intricatum* Boiss), are very suitable for hosting web-weaving spiders that catch many adult whiteflies [75]. The authors suggested that inclusion of these shrub species supporting spiders in hedgerows between greenhouses can reduce immigration of thrips and whiteflies into the greenhouses and, therefore, also reduce infection of the crops by viruses transmitted by these pests. These spider-hosting hedgerows could thus act as a kind of phytosanitary barriers for pests and insect-transmitted viral diseases [75]. Other native perennial plants can also support predators and parasitoids of thrips and whiteflies, but some of these plants are also good hosts of the pests themselves [36]. The influx of these pests in greenhouses might be reduced by selecting plants that produce only low to medium pest levels but still support parasitoids and predators to control these pests [36]. In order to optimize pest suppression around greenhouses, plant species with specific functional traits should be selected that support specific target natural enemies. For example, aphid control could be enhanced by using plants species that provide nectar or pollen for the natural enemies of aphids, such as syrphids, lacewings, ladybeetles, and aphid parasitoids. Adult syrphids need plants with accessible nectar for their survival, which depends on the depths of the flowers [76]. Some species of ladybeetles and lacewings are attracted to flowering plants with a specific ultra-violet pattern [70]. Predatory bugs of the genus *Orius* are important natural enemies of thrips and need pollen and nectar for their survival on plants in the absence of prey [77,78]. Besides food sources, they need soft plant tissue for oviposition, and the preferred plants for oviposition may differ from those plants that supply pollen and nectar [78]. Most plant species that provide pollen for predators are also suitable for pollen-feeding thrips species, like western flower thrips and onion thrips [37]. However, there are large differences in the suitability of different pollen species for thrips [79]. It might thus be possible to select plant species that have a low suitability for thrips and high suitability for *Orius* predators, which would be ideal for maximising thrips suppression near greenhouses. Field observations on wild plants in Turkey indicate that Paterson’s curse (*Echium plantagineum* L.), dwarf nettle (*Urtica urens* L.), field marigold (*Calendula arvensis* Vaill.), and henbit deadnettle (*Lamium amplexicaule* L.) are good candidates for this purpose [80]. Certain zoophytophagous bugs of the family Miridae are important predators for control of whiteflies, aphids, thrips, and lepidopteran pests [81,82,83,84,85]. High densities of these predators near greenhouses might also contribute to pest management of greenhouse pests on wild vegetation. Because of the plant feeding habits of these predators and their need to oviposit inside plant tissue, they prefer hairy plants with soft plant tissue, like *Geranium* species, pot marigold (*Calendula officinalis* L.), or European black nightshade (*Solanum nigrum* L.) [86,87]. Providing such plants in the greenhouse vicinity might boost predator densities and enhance conservation biological control of greenhouse pests in the vegetation surrounding the greenhouse, thereby reducing the influx into greenhouses [55]. Here, too, caution is needed because some plant species may be good hosts for pests, such as *S. nigrum* is for *T. absoluta* [88]. Plants can also provide certain micro-habitats or structures that function as refugia for natural enemies, such as tufts of plant hairs in the leaf vein axils that function as domatia for predatory mites [89]. In addition, habitats near greenhouses can also provide shelter for natural enemies, and this can be optimized to increase natural enemy densities. For example, lacewing densities might be increased by providing overwintering chambers with straw [90], and mulched plant residuals near greenhouses can increase spider and predatory beetle densities [91]. Plants do not only supply refuges and pollen and nectar, but they can also provide non-pest prey to natural enemies. Plants like *Achillea millefolium* L. and *Centaurea jacea* L. host specific non-pest aphids that boost aphid predator densities (Lambion personal observations). Thus, to support different species of natural enemies, mixtures of plant species with different functional traits will be needed. These plants should preferably be native to support the positive effects of indigenous fauna.

### 4.3. Retaining Pest Species with Preferred Host Plants

Several studies suggest the use of trap crops to lure pests away from the target crop [8]. For example, field studies in Italy showed that flower strips near tomato crops harboured high densities of tarnished plant bugs and stink bugs, but the densities and damage in the crop near the flower strips were lower than in control crops without flower strips [92]. Others studies showed similar effects by using specific trap plants to reduce densities of tarnished plant bugs in adjacent crops [39,93]. However, a preferred host plant is often a better host plant, in which case the benefit of using trap plants may be short-lived: after establishing a population on these plants, populations will grow faster than in the crop and may then spill over to the crop. The use of preferred host plants of pests might reduce pest densities when the trap plants are also good hosts for the natural enemies of the target pest [94], resulting in a kind of attract-and-kill-system. But whether such a system would work in the long term is uncertain as well; it may result in the selection of herbivores that avoid these trap plants and prefer the crop. Thus, using plants near greenhouses that are attractive for pests might be a risky approach and not suitable for reducing the influx of greenhouse pests in the long run.

## 5. Biodiversity in Greenhouses

Biodiversity may not only be useful outside greenhouses but can also be utilized with various methods inside greenhouses to conserve and augment natural enemy populations [95,96]. A well-proven implemented method is the use of banker plants, which are specific plants that produce (“banker”) natural enemies with alternative prey/hosts or alternative food that poses no threat for the crop (Figure 2) [97,98]. A widely applied system in greenhouse crops has been the use of monocotyledonous plants with cereal aphids that serve as alternative hosts for parasitoids of aphid pests in dicotyledonous crops [99]. Non-crop plants may also be introduced to provide nectar and pollen. This can even be beneficial for crops that produce nectar and pollen themselves, such as sweet pepper. Adding flowering sweet alyssum and coriander to this crop was shown to increase syrphid reproduction, which is beneficial for aphid control [100]. Likewise, pollen-producing plants, such as ornamental pepper or marigold, can be used to support and increase pollen-feeding predators, such as *Orius* spp. [101,102] and mirids. Other non-crop plants can be used to provide shelter, like domatia for predatory mites [103], or function as oviposition plants for predatory bugs [104]. A method derived from adding non-crop plants that provide food to natural enemies is the direct supply of alternative food for generalist predators. Various alternative food types, such as pollen, sterilized moth eggs, shrimp cysts, and astigmatid mites, are now commercially produced for this objective [95]. However, some of these foods are also edible for omnivorous thrips species, and caution is needed when initial thrips densities are relatively high compared to predator densities [105,106,107]. Some studies have shown potential for the enhancement of predatory mite establishment through the provision of artificial domatia [108,109], but to our knowledge, this has not been applied commercially so far.

Pest control in greenhouses might also be enhanced by diversifying cropping systems. Pest populations are known to develop slower in mixed cropping systems than in monocultures, which can be the result of both disruption of the host-plant locating ability of pests and an improved performance of the natural enemies of these pests [110,111]. However, mixing crops in greenhouses is not very popular for economic reasons because each crop needs its own climatic conditions, harvest, and packaging systems. Nevertheless, growers with organic cropping systems often produce a more varied package of products. Biodiversity in greenhouses might also be linked with biodiversity outside greenhouses, e.g., for attracting and retaining natural enemies that enter greenhouses from the outdoor vegetation. Planting perennial plants in greenhouses is an interesting approach for diversifying cropping systems because it offers possibilities to maintain natural enemy populations when greenhouses are cleaned during crop rotations [112]. Compared to outdoor crops, flower strips may function well in greenhouses because they are better protected against climate extremes. They can not only support the natural enemies that enter greenhouses but also those that are released for pest control. However, little is known about the costs and benefits of using such flower strips in greenhouses, and this clearly needs further studies.

## 6. Conclusions and Future Recommendations

There is an ongoing debate about whether biodiversity and plant diversity around greenhouses is a potential risk or benefit for pest and disease management in greenhouses. The results of this review on the effects of increased plant diversity as a strategy for more sustainable greenhouse production systems is summarized in Figure 2. We show here that most greenhouse pests in temperate climates are of exotic origin and probably have settled permanently in greenhouses. New infestations of greenhouses by these exotic pests often occur through transportation of plant material or through migration from greenhouse to greenhouse, whereas invasions from plants in the vicinity of greenhouses are limited. Furthermore, most indigenous pests, such as aphids, spider mites, and some thrips species, can travel long distances. This all suggests that maintaining low diversity of surrounding vegetation will not prevent pests from entering greenhouses. Nevertheless, there are certain risks associated with higher plant diversity around greenhouses, such as infections by viruses transmitted by insects or increased influx of hyperparasitoids, and these need to be considered when designing cropping systems with higher plant diversity. In our opinion, however, these potential risks will often not outweigh the many benefits of biodiversity around greenhouses, particularly for organic cropping systems or crops with a minimum use of pesticides.

These benefits are: (1) biodiversity around greenhouses can contribute substantially to pest management in greenhouses by facilitating the influx of natural enemies that suppress indigenous and exotic pests inside greenhouses. (2) Biodiversity around greenhouses can support the influx of insects that contribute to the pollination of some greenhouse crops. (3) Plant diversity around greenhouses can play an important role for some pest species through reducing their densities outside greenhouses with natural enemies. (4) Vegetation around greenhouses can function as a trap for greenhouse pests and prevent migration to neighbouring greenhouses, but this may only function well if these pests are also controlled by natural enemies on those plants in order to prevent a spill-over to the greenhouse crops. How the influx of natural enemies to greenhouses and pest suppression of greenhouse pests near greenhouses by natural enemies can be promoted by specific plant communities, plant characteristics, and habitats while minimising risks of promoting greenhouse pests, virus transmission, or hyperparasitoids needs further studies. It also requires a better understanding of the underlying processes linking biodiversity with pest management. Nevertheless, there are various general measures that growers can already apply to enhance pest control with biodiversity at different spatial scales, which are summarised in Table 2.

Optimising ecosystems in and around greenhouses for pest suppression through these elements of biodiversity will strongly depend on climate, landscape, pest and natural enemy diversity and densities, as well as the greenhouse cropping system. Thus, tailormade approaches are needed for each situation. Yet, we hope that our study will further stimulate the exploration of using biodiversity for pest control in greenhouses, which has largely been ignored. Traditionally, augmentative biological control, which is the release of mass-produced natural enemies [113], was clearly distinguished from conservation biological control, but the current supply of alternative food for natural enemies inside greenhouses is a first step into integrating these types of control. In our opinion, the use of plant diversity inside and outside greenhouses is a logical next step. We think it will be particularly interesting to further study how such biodiversity inside and outside greenhouses can be linked to enhancement of biological pest control with both released and naturally occurring species of natural enemies.

## Figures and Tables

**Table 1:** Common pests in Northern European greenhouses [22,23] (and personal observations).

Pest Group and Order	Common Name	Scientific Name	Origin
Thrips	Western flower thrips	*Frankliniella occidentalis* Pergande	Exotic, USA
(Thysanoptera)	Onion thrips	*Trips tabaci* Lindeman	Indigenous
	Japanese flower thrips	*Thrips setosus* Moulton	Exotic, Japan
	Poinsettia thrips	*Echinothrips americanus* Morgan	Exotic, USA
	Orchid thrips	*Chaetanaphothrips* *Orchidii* (Moulton)	Exotic, tropical
	Vanda thrips	*Dichromothrips corbetti* (Priesner)	Exotic, tropical
	Palm thrips	*Parthenothrips dracaenae* (Heeger)	Exotic, tropical
	Banded greenhouse thrips	*Hercinothrips femoralis* (Reuter)	Exotic, tropical
	Tobacco thrips	*Thrips parvispinus* (Karny)	Exotic, Asia
	Rose thrips	*Thrips fuscipennis* Haliday	Indigenous
	European flower thrips	*Frankliniella intonsa* (Trybom)	Indigenous
Whiteflies	Greenhouse whitefly	*Trialeurodes vaporariorum* (Westwood)	Exotic, South America
(Hemiptera)	Tobacco whitefly	*Bemisia tabaci* (Gennadius)	Exotic, Mediterranean
Aphids	Green peach aphid	*Myzus persicae* (Sulzer)	Indigenous
(Hemiptera)	Foxglove aphid	*Aulacorthum solani* (Kaltenbach)	Indigenous
	Potato aphid	*Macrosiphum euphorbiae* (Thomas)	Indigenous
	Cotton aphid	*Aphis gosyppii* Glover	Indigenous
Mealybugs	Citrus mealybug	*Planacoccus citri* (Risso)	Exotic, Mediterranean
(Hemiptera)	Long-tailed mealybug	*Pseudococcus longispinus* (Targioni-Tozzetti)	Exotic, Mediterranean
	Obscure mealybug	*Pseudococcus viburni* (Signoret)	Exotic, South America
Armoured scales	Boisduval scale	*Diaspis boisduvalii* Signoret	Exotic, tropical
(Hemiptera)	Rose scale	*Aulacaspis rosae* (Bouché)	Exotic, subtropical
Spider mites	Two-spotted spider mite	*Tetranychus urticae* Koch	Indigenous
(Trombidiformes)			
Tarsonemid mites	Bulb scale mite	*Steneotarsonemus laticeps* (Halbert)	Exotic
(Trombidiformes)	Broad mite	*Polyphagotarsonemus latus* (Banks)	Exotic, tropical, subtropical
Eriophyid mites (Trombidiformes)	Tomato russet mite	*Aculops lycopersici* (Tryon)	Exotic, Mediterranean
Bugs		*Nesidiocoris tenuis* (Reuter)	Exotic, Mediterranean
(Hemiptera)	Southern green stink bug	*Nezara viridula* (Linnaeus)	Exotic, Mediterranean
	Tarnished plant bug	*Lygus rugulipennis* Poppius	Indigenous
	Common nettle bug	*Liocoris tripustulatus* (Fabricius)	Indigenous
Caterpillars	South American tomato pinworm	*Tuta absoluta* (Meyrick)	Exotic, South-America
(Lepidoptera)	Golden twin-spot moth	*Chrysodeixis chalcitis* (Esper)	Exotic, Mediterranean
	Southern European marshland pyralid	*Duponchelia fovealis* Zeller	Exotic, Mediterranean
	Cabbage leafroller	*Clepsis spectrana* (Treitschke)	Indigenous
	Carnation tortrix	*Cacoecimorpha pronubana* (Hübner)	Indigenous
Flies and midges	Cabbage fly	*Delia radicum* (Linnaeus)	Indigenous
(Diptera)	Lyprauta	*Lyprauta chacoensis* (Edwards) *Lyprauta cambria* Chandler & Pijnakker	Exotic, tropical

**Table 2:** Measures inside and outside greenhouses to support biological pest control through functional biodiversity.

Inside Greenhouses	Adjacent to Greenhouses	Landscape around Greenhouses
Releases of mass-produced natural enemies (augmentative biological control)	flowering strips and shrubs with year-round successive flowering periods that support natural enemies (conservation biological control) *	Connection with ecological structures like hedgerows
Providing supplemental food sources for natural enemies (mites, pollen, eggs)	Shrubs and plants that are hosts for non-pest herbivores, which serve as food for natural enemies	
Banker plants, oviposition plants for natural enemies	Shelters for overwintering of natural enemies: woody shrubs, dead wood, nest structures	
Strips of flowering plants		

* These mixtures should not contain plants that are highly attractive for greenhouse pests unless they are also highly attractive for the natural enemies of these pests.

## Data Availability

Not applicable.

## References

[1] Cardinale B.J., Duffy J.E., Gonzalez A., Hooper D.U., Perrings C., Venail P., Narwani A., Mace G.M., Tilman D., Wardle D.A. (2012). Biodiversity loss and its impact on humanity. Nature.

[2] Barbosa P. (1998). Conservation Biological Control.

[3] Letourneau D.K., Jedlicka J.A., Bothwell S.G., Moreno C.R. (2009). Effects of natural enemy biodiversity on the suppression of arthropod herbivores in terrestrial ecosystems. Annu. Rev. Ecol. Evol. Syst..

[4] Gurr G.M., Wratten S.D., Landis D.A., You M.S. (2017). Habitat management to suppress pest populations: Progress and prospects. Annu. Rev. Entomol..

[5] Bianchi F., Booij C.J.H., Tscharntke T. (2006). Sustainable pest regulation in agricultural landscapes: A review on landscape composition, biodiversity and natural pest control. Proc. R. Soc. Lond. Ser. B Biol. Sci..

[6] Jonsson M., Wratten S.D., Landis D.A., Gurr G.M. (2008). Recent advances in conservation biological control of arthropods by arthropods. Biol. Control.

[7] Begg G.S., Cook S.M., Dye R., Ferrante M., Franck P., Lavigne C., Lovei G.L., Mansion-Vaquie A., Pell J.K., Petit S. (2017). A functional overview of conservation biological control. Crop Prot..

[8] Letourneau D.K., Armbrecht I., Rivera B.S., Lerma J.M., Carmona E.J., Daza M.C., Escobar S., Galindo V., Gutierrez C., Lopez S.D. (2011). Does plant diversity benefit agroecosystems? A synthetic review. Ecol. Appl..

[9] Gurr G.M., Wratten S.D., Luna J.M. (2003). Multi-function agricultural biodiversity: Pest management and other benefits. Basic Appl. Ecol..

[10] Rosenheim J.A. (1998). Higher-order predators and the regulation of insect herbivore populations. Annu. Rev. Entomol..

[11] Sullivan D.J., Völkl W. (1999). Hyperparasitism: Multitrophic ecology and behavior. Annu. Rev. Entomol..

[12] Martin E.A., Dainese M., Clough Y., Baldi A., Bommarco R., Gagic V., Garratt M.P.D., Holzschuh A., Kleijn D., Kovacs-Hostyanszki A. (2019). The interplay of landscape composition and configuration: New pathways to manage functional biodiversity and agroecosystem services across Europe. Ecol. Lett..

[13] Chaplin-Kramer R., O’Rourke M.E., Blitzer E.J., Kremen C. (2011). A meta-analysis of crop pest and natural enemy response to landscape complexity. Ecol. Lett..

[14] Holland J.M., Bianchi F., Entling M.H., Moonen A.C., Smith B.M., Jeanneret P. (2016). Structure, function and management of semi-natural habitats for conservation biological control: A review of European studies. Pest. Manag. Sci..

[15] Hallmann C.A., Sorg M., Jongejans E., Siepel H., Hofland N., Schwan H., Stenmans W., Müller A., Sumser H., Hörren T. (2017). More than 75 percent decline over 27 years in total flying insect biomass in protected areas. PLoS ONE.

[16] Sanchez-Bayo F., Wyckhuys K.A.G. (2019). Worldwide decline of the entomofauna: A review of its drivers. Biol. Conserv..

[17] Stanghellini C., Van’t Ooster B., Heuvelink E. (2019). Greenhouse Horticulture: Technology for Optimal Crop Production.

[18] Kirk W.D.J., Terry L.I. (2003). The spread of the western flower thrips *Frankliniella occidentalis* (Pergande). Agric. For. Entomol..

[19] Cao L.J., Gao Y.F., Gong Y.J., Chen J.C., Chen M., Hoffmann A., Wei S.J. (2019). Population analysis reveals genetic structure of an invasive agricultural thrips pest related to invasion of greenhouses and suitable climatic space. Evol. Appl..

[20] Desneux N., Wajnberg E., Wyckhuys K.A.G., Burgio G., Arpaia S., Narvaez-Vasquez C.A., Gonzalez-Cabrera J., Ruescas D.C., Tabone E., Frandon J. (2010). Biological invasion of European tomato crops by *Tuta absoluta*: Ecology, geographic expansion and prospects for biological control. J. Pest. Sci..

[21] Dalmon A., Halkett F., Granier M., Delatte H., Peterschmitt M. (2008). Genetic structure of the invasive pest *Bemisia tabaci*: Evidence of limited but persistent genetic differentiation in glasshouse populations. Heredity.

[22] Gullino M.L., Albajes R., Nicot P. (2020). Integrated Pest and Disease Management in Greenhouse Crops.

[23] Van Driesche R.G., Heinz K.M., Heinz K.M., Van Driesche R.G., Parrella M.P. (2004). An overview of biological control in protected culture. Biocontrol in Protected Culture.

[24] Vierbergen G., Marullo R., Mound L. (2002). Occurrence of glasshouse Thysanoptera in the open in the Netherlands. The 7th International Symposium on Thysanoptera, Calabria, Italy, 2–7 July 2001.

[25] Sampson C., Bennison J., Kirk W.D.J. (2021). Overwintering of the western flower thrips in outdoor strawberry crops. J. Pest. Sci..

[26] Makra L., Bodnar K., Fulop A., Orosz S., Szenasi A., Csepe Z., Jenser G., Tusnady G., Magyar D. (2018). The first record of subtropical insects (Thysanoptera) in central Europe: Long-distance transport of airborne thrips, applying three-dimensional backward trajectories. Agric. For. Entomol..

[27] Lewis T. (1964). The weather and mass flights of Thysanoptera. Ann. Appl. Biol..

[28] Johnson C.G. (1969). Migration and Dispersal of Insects by Flight.

[29] Jenser G. (1973). Observations on the autumn mass flight of *Frankliniella intonsa* Trybom (Thysanoptera, Thripidae). Acta Phytopathol. Acad. Sci. Hung..

[30] Bell J.R., Bohan D.A., Shaw E.M., Weyman G.S. (2005). Ballooning dispersal using silk: World fauna, phylogenies, genetics and models. Bull. Entomol. Res..

[31] Clotuche G., Navajas M., Mailleux A.C., Hance T. (2013). Reaching the ball or missing the flight? Collective dispersal in the two-spotted spider mite *Tetranychus urticae*. PLoS ONE.

[32] Müller C.B., Williams I.S., Hardie J. (2001). The role of nutrition, crowding and interspecific interactions in the development of winged aphids. Ecol. Entomol..

[33] Kim J., Kwon M. (2019). Population dynamics of aphid species in Korean seed potato cultivation area over four decades. Entomol. Res..

[34] Webb S.E., Hochmuth R.C. (2016). Vegetable insect identification and management. Florida Greenhouse Vegetable Production Handbook.

[35] Knapp M., Palevsky E., Rapisarda C., Gullino M.L., Albajes R., Nicot P.C. (2020). Insect and mite pests. Integrated Pest and Disease Management in Greenhouse Crops.

[36] Rodriguez E., Gonzalez M., Paredes D., Campos M., Benitez E. (2018). Selecting native perennial plants for ecological intensification in Mediterranean greenhouse horticulture. Bull. Entomol. Res..

[37] Kirk W.D.J. (1985). Pollen-feeding and the host specificity and fecundity of flower thrips (Thysanoptera). Ecol. Entomol..

[38] Li S., Jaworski C.C., Hatt S., Zhang F., Desneux N., Wang S. (2021). Flower strips adjacent to greenhouses help reduce pest populations and insecticide applications inside organic commercial greenhouses. J. Pest. Sci..

[39] Easterbrook M.A., Tooley J.A. (1999). Assessment of trap plants to regulate numbers of the European tarnished plant bug, *Lygus rugulipennis*, on late-season strawberries. Entomol. Exp. Appl..

[40] Ondiaka S., Migiro L., Rur M., Birgersson G., Porcel M., Ramert B., Tasin M. (2016). Sunflower as a trap crop for the European tarnished plant bug (*Lygus rugulipennis*). J. Appl. Entomol..

[41] Winkler K., Waeckers F.L., Termorshuizen A.J., van Lenteren J.C. (2010). Assessing risks and benefits of floral supplements in conservation biological control. BioControl.

[42] Balzan M.V., Bocci G., Moonen A.C. (2016). Utilisation of plant functional diversity in wildflower strips for the delivery of multiple agroecosystem services. Entomol. Exp. Appl..

[43] Parrella G., Gognalons P., Gebre-Selassie K., Vovlas C., Marchoux G. (2003). An update of the host range of tomato spotted wilt virus. J. Plant. Pathol..

[44] Stobbs L.W., Broadbent A.B., Allen W.R., Stirling A.L. (1992). Transmission of Tomato Spotted Wilt Virus by the western flower thrips to weeds and native plants found in southern Ontario. Plant Dis..

[45] Rotenberg D., Jacobson A.L., Schneweis D.J., Whiffleld A.E. (2015). Thrips transmission of tospoviruses. Curr. Opin. Virol..

[46] Ng J.C.K., Falk B.W. (2006). Virus-vector interactions mediating nonpersistent and semipersistent transmission of plant viruses. Annu. Rev. Phytopathol..

[47] Lecoq H., Smith H.G., Barker H. (1999). Epidemiology of Cucurbit aphid-borne yellows virus. The Luteoviridae.

[48] Navas-Castillo J., Fiallo-Olive E., Sanchez-Campos S. (2011). Emerging virus diseases transmitted by whiteflies. Annu. Rev. Phytopathol..

[49] Velasco L., Simon B., Janssen D., Cenis J.L. (2008). Incidences and progression of tomato chlorosis virus disease and tomato yellow leaf curl virus disease in tomato under different greenhouse covers in southeast Spain. Ann. Appl. Biol..

[50] Albrecht M., Kleijn D., Williams N.M., Tschumi M., Blaauw B.R., Bommarco R., Campbell A.J., Dainese M., Drummond F.A., Entling M.H. (2020). The effectiveness of flower strips and hedgerows on pest control, pollination services and crop yield: A quantitative synthesis. Ecol. Lett..

[51] Gerling D., Alomar O., Arno J. (2001). Biological control of *Bemisia tabaci* using predators and parasitoids. Crop. Prot..

[52] Castañé C., Alomar O., Goula M., Gabarra R. (2004). Colonization of tomato greenhouses by the predatory mirid bugs *Macrolophus caliginosus* and *Dicyphus tamaninii*. Biol. Control.

[53] Gabarra R., Alomar O., Castañé C., Goula M., Albajes R. (2004). Movement of greenhouse whitefly and its predators between in- and outside of Mediterranean greenhouses. Agric. Ecosyst. Environ..

[54] Ingegno B.L., Pansa M.G., Tavella L. (2009). Tomato colonization by predatory bugs (Heteroptera: Miridae) in agroecosystems of NW Italy. IOBC WPRS Bull..

[55] Perdikis D., Fantinou A., Lykouressis D. (2011). Enhancing pest control in annual crops by conservation of predatory Heteroptera. Biol. Control.

[56] Lambion J. (2011). Functional biodiversity in southern France: A method to enhance predatory mirid bug populations. Acta Hortic..

[57] Bosco L., Tavella L. (2013). Distribution and abundance of species of the genus *Orius* in horticultural ecosystems of northwestern Italy. Bull. Insectol..

[58] Postic E., Le Ralec A., Buchard C., Granado C., Outreman Y. (2020). Variations in community assemblages and trophic networks of aphids and parasitoids in protected crops. Ecosphere.

[59] Naselli M., Biondi A., Garzia G.T., Desneux N., Russo A., Siscaro G., Zappala L. (2017). Insights into food webs associated with the South American tomato pinworm. Pest. Manag. Sci..

[60] Grosman A., Bloemhard C. (2013). Nieuwe Sluipwespen Tegen Turkse Mot, Chrysodeixis Chalcites, in Paprika.

[61] Abe Y., Takeuchi T., Tokumaru S., Kamata J. (2005). Comparison of the suitability of three pest leafminers (Diptera: Agromyzidae) as hosts for the parasitoid *Dacnusa sibirica* (Hymenoptera: Bracenidae). Eur. J. Entomol..

[62] Woets J., van der Linden A. (1982). On the occurrence of *Opius pallipes* Wesmael and *Dacnusa sibirica* Telenga (Braconidae) in cases of natural control of the tomato leafminer *Liriomyza bryoniae* Kalt. (Agromyzidae) in some large greenhouses in the Netherlands. Med. Fac. Landbouw. Rijksuniv. Gent.

[63] Chailleux A., Mohl E.K., Alves M.T., Messelink G.J., Desneux N. (2014). Natural enemy-mediated indirect interactions among prey species: Potential for enhancing biocontrol services in agroecosystems. Pest. Manag. Sci..

[64] Yu H.L., Zhang Y.J., Wu K.M., Wyckhuys K.A.G., Guo Y.Y. (2009). Flight potential of *Microplitis mediator*, a parasitoid of various lepidopteran pests. BioControl.

[65] Winkler K., Wäckers F., Bukovinszkine-Kiss G., van Lenteren J. (2006). Sugar resources are vital for *Diadegma semiclausum* fecundity under field conditions. Basic Appl. Ecol..

[66] Heimpel G.E. (2019). Linking parasitoid nectar feeding and dispersal in conservation biological control. Biol. Control.

[67] Araj S.E., Wratten S., Lister A., Buckley H. (2009). Adding floral nectar resources to improve biological control: Potential pitfalls of the fourth trophic level. Basic Appl. Ecol..

[68] Bloemhard C.M.J., van der Wielen M., Messelink G.J. (2014). Seasonal abundance of aphid hyperparasitoids in organic greenhouse crops in the Netherlands. IOBC WPRS Bull..

[69] Dong Z.K., Men X.Y., Liu S., Zhang Z.Y. (2019). Food web structure of parasitoids in greenhouses is affected by surrounding landscape at different spatial scales. Sci. Rep..

[70] Hatt S., Uytenbroeck R., Lopes T., Mouchon P., Osawa N., Piqueray J., Monty A., Francis F. (2019). Identification of flower functional traits affecting abundance of generalist predators in perennial multiple species wildflower strips. Arthropod Plant. Interact..

[71] Martin C.D., Fountain M.T., Brown M.J.F. (2019). Varietal and seasonal differences in the effects of commercial bumblebees on fruit quality in strawberry crops. Agric. Ecosyst. Environ..

[72] Dunn L., Lequerica M., Reid C.R., Latty T. (2020). Dual ecosystem services of syrphid flies (Diptera: Syrphidae): Pollinators and biological control agents. Pest. Manag. Sci..

[73] Xu X.M., Jay C.N., Fountain M.T., Linka J., Fitzgerald J.D. (2014). Development and validation of a model forecasting the phenology of European tarnished plant bug *Lygus rugulipennis* in the UK. Agric. For. Entomol..

[74] Pansa M.G., Guidone L., Tavella L. (2012). Distribution and abundance of nymphal parasitoids of *Lygus rugulipennis* and *Adelphocoris lineolatus* in northwestern Italy. Bull. Insectol..

[75] Cotes B., Gonzalez M., Benitez E., De Mas E., Clemente-Orta G., Campos M., Rodriguez E. (2018). Spider communities and biological control in native habitats surrounding greenhouses. Insects.

[76] Van Rijn P.C.J., Wäckers F.L. (2016). Nectar accessibility determines fitness, flower choice and abundance of hoverflies that provide natural pest control. J. Appl. Ecol..

[77] Cocuzza G.E., DeClercq P., VandeVeire M., DeCock A., Degheele D., Vacante V. (1997). Reproduction of *Orius laevigatus* and *Orius albidipennis* on pollen and *Ephestia kuehniella* eggs. Entomol. Exp. Appl..

[78] Pumariño L., Alomar O., Lundgren J.G. (2012). Effects of floral and extrafloral resource diversity on the fitness of an omnivorous bug, *Orius insidiosus*. Entomol. Exp. Appl..

[79] Hulshof J., Ketoja E., Vänninen I. (2003). Life history characteristics of *Frankliniella occidentalis* on cucumber leaves with and without supplemental food. Entomol. Exp. Appl..

[80] Atakan E., Tunc I. (2010). Seasonal abundance of hemipteran predators in relation to western flower thrips *Frankliniella occidentalis* (Thysanoptera: Thripidae) on weeds in the eastern Mediterranean region of Turkey. Biocontrol Sci. Technol..

[81] Riudavets J., Castañé C. (1998). Identification and evaluation of native predators of *Frankliniella occidentalis* (Thysanoptera: Thripidae) in the Mediterranean. Environ. Entomol..

[82] Montserrat M., Albajes R., Castañé C. (2000). Functional response of four Heteropteran predators preying on greenhouse whitefly (Homoptera: Aleyrodidae) and western flower thrips (Thysanoptera: Thripidae). Environ. Entomol..

[83] Urbaneja A., Monton H., Molla O. (2009). Suitability of the tomato borer *Tuta absoluta* as prey for *Macrolophus pygmaeus* and *Nesidiocoris tenuis*. J. Appl. Entomol..

[84] Ingegno B.L., Bodino N., Leman A., Messelink G.J., Tavella L. (2017). Predatory efficacy of *Dicyphus errans* on different prey. Acta Hortic..

[85] Messelink G.J., Bloemhard C.M.J., Hoogerbrugge H., van Schelt J., Ingegno B.L., Tavella L. (2015). Evaluation of mirid predatory bugs and release strategy for aphid control in sweet pepper. J. Appl. Entomol..

[86] Ingegno B.L., Goula M., Navone P., Tavella L. (2008). Distribution and host plants of the genus *Dicyphus* in the Alpine valleys of NW Italy. Bull. Insectol..

[87] Ingegno B.L., Pansa M.G., Tavella L. (2011). Plant preference in the zoophytophagous generalist predator *Macrolophus pygmaeus* (Heteroptera: Miridae). Biol. Control.

[88] Ingegno B.L., Candian V., Psomadelis I., Bodino N., Tavella L. (2017). The potential of host plants for biological control of *Tuta absoluta* by the predator *Dicyphus errans*. Bull. Entomol. Res..

[89] Walter D.E. (1996). Living on leaves: Mites, tomenta, and leaf domatia. Annu. Rev. Entomol..

[90] Thierry D., Rat-Morris E., Caldumbide C. (2002). Selective attractivity of artificial overwintering chambers for the common green lacewing species of the *Chrysoperla carnea* (Stephens) complex in western Europe (Neuroptera: Chrysopidae). Acta Zool. Acad. Sci. Hung..

[91] Xu Q.C., Fujiyama S., Xu H.L. (2012). Pest control by enriching natural enemies under artificial habitat management along sidewalls of greenhouse in organic farming systems. J. Food Agric. Environ..

[92] Balzan M.V., Moonen A.C. (2014). Field margin vegetation enhances biological control and crop damage suppression from multiple pests in organic tomato fields. Entomol. Exp. Appl..

[93] Swezey S.L., Nieto D.J., Hagler J.R., Pickett C.H., Bryer J.A., Machtley S.A. (2013). Dispersion, distribution, and movement of *Lygus* spp. (Hemiptera: Miridae) in trap-cropped organic strawberries. Environ. Entomol..

[94] Hagler J.R., Nieto D.J., Machtley S.A., Swezey S.L. (2020). Predator demographics and dispersal in alfalfa trap-cropped strawberry. Entomol. Exp. Appl..

[95] Messelink G.J., Bennison J., Alomar O., Ingegno B.L., Tavella L., Shipp L., Palevsky E., Wäckers F.L. (2014). Approaches to conserving natural enemy populations in greenhouse crops: Current methods and future prospects. BioControl.

[96] Pijnakker J., Vangansbeke D., Duarte M., Moerkens R., Wäckers F.L. (2020). Predators and parasitoids-in-first: From inundative releases to preventative biological control in greenhouse crops. Front. Sustain. Food Syst..

[97] Huang N.X., Enkegaard A., Osborne L.S., Ramakers P.M.J., Messelink G.J., Pijnakker J., Murphy G. (2011). The banker plant method in biological control. Crit. Rev. Plant Sci..

[98] Frank S.D. (2010). Biological control of arthropod pests using banker plant systems: Past progress and future directions. Biol. Control.

[99] Bennison J. (1992). Biological control of aphids on cucumbers use of open rearing systems or ‘banker plants’ to aid establishment of *Aphidius matricariae* and *Aphidoletes aphidimyza*. Med. Fac. Landbouw. Rijksuniv. Gent.

[100] Pineda A., Marcos-García M.A. (2008). Use of selected flowering plants in greenhouses to enhance aphidophagous hoverfly populations (Diptera: Syrphidae). Ann. Soc. Entomol. Fr..

[101] Waite M.O., Scott-Dupree C.D., Brownbridge M., Buitenhuis R., Murphy G. (2014). Evaluation of seven plant species/cultivars for their suitability as banker plants for *Orius insidiosus* (Say). BioControl.

[102] Zhao J., Guo X.J., Tan X.L., Desneux N., Zappala L., Zhang F., Wang S. (2017). Using *Calendula officinalis* as a floral resource to enhance aphid and thrips suppression by the flower bug *Orius sauteri* (Hemiptera: Anthocoridae). Pest. Manag. Sci..

[103] Parolin P., Bresch C., Ruiz G., Desneux N., Poncet C. (2013). Testing banker plants for biological control of mites on roses. Phytoparasitica.

[104] Kakimoto K., Inoue H., Yamaguchi T., Fukamachi S., Shima K., Taguchi Y., Saiki Y., Ohno K. (2007). Simultaneous release of *Orius strigicollis* (Poppius) eggs and adults to improve its establishment in greenhouses. Jpn. J. Appl. Entomol. Zool..

[105] Van Rijn P.C.J., van Houten Y.M., Sabelis M.W. (2002). How plants benefit from providing food to predators even when it is also edible to herbivores. Ecology.

[106] Leman A., Messelink G.J. (2015). Supplemental food that supports both predator and pest: A risk for biological control?. Exp. Appl. Acarol..

[107] Vangansbeke D., Nguyen D.T., Audenaert J., Verhoeven R., Gobin B., Tirry L., De Clercq P. (2016). Supplemental food for *Amblyseius swirskii* in the control of thrips: Feeding friend or foe?. Pest. Manag. Sci..

[108] Loughner R., Nyrop J., Wentworth K., Sanderson J. (2011). Effects of supplemental pollen and fibers on canopy abundance of *Amblyseius swirskii*. IOBC WPRS Bull..

[109] Bresch C., Carlesso L., Suay R., Van Oudenhove L., Touzeau S., Fatnassi H., Ottenwaelder L., Paris B., Poncet C., Mailleret L. (2019). In search of artificial domatia for predatory mites. Biocontrol Sci. Technol..

[110] Manandhar R., Wang K.H., Hooks C.R.R., Wright M.G. (2017). Effects of strip-tilled cover cropping on the population density of thrips and predatory insects in a cucurbit agroecosystem. J. Asia Pac. Entomol..

[111] Letourneau D.K., Altieri M.A. (1983). Abundance patterns of a predator, *Orius tristicolor* (Hemiptera, Anthocoridae), and its prey, *Frankliniella occidentalis* (Thysanoptera, Thripidae)—Habitat attraction in polycultures versus monocultures. Environ. Entomol..

[112] Lambion J., van Rijn P. (2021). Flower Strips: A tool for Pest Control in Greenhouses. Organic E-Prints Document 38705. p. 4. https://orgprints.org/id/eprint/38705/1/FlowerStrips_GreenResilient.pdf.

[113] Bale J.S., van Lenteren J.C., Bigler F. (2008). Biological control and sustainable food production. Philos. Trans. R. Soc. B.

